# Reconstruction of Mini‐Hollow Polyhedron Mn_2_O_3_ Derived from MOFs as a High‐Performance Lithium Anode Material

**DOI:** 10.1002/advs.201500185

**Published:** 2015-08-25

**Authors:** Kangzhe Cao, Lifang Jiao, Hang Xu, Huiqiao Liu, Hongyan Kang, Yan Zhao, Yongchang Liu, Yijing Wang, Huatang Yuan

**Affiliations:** ^1^ Key Laboratory of Advanced Energy Materials Chemistry (MOE)Collaborative Innovation Center of Chemical Science and Engineering (Tianjin)Nankai UniversityTianjin300071P. R. China

**Keywords:** anode materials, Mn_2_O_3_ electrodes, Li‐ion batteries, mini‐hollow structures, metal‐organic frameworks

## Abstract

**A mini‐hollow polyhedron Mn_2_O_3_** **is used as the anode material** for lithium‐ion batteries. Benefiting from the small interior cavity and intrinsic nanosize effect, a stable reconstructed hierarchical nanostructure is formed. It has excellent energy storage properties, exhibiting a capacity of 760 mAh g^−1^ at 2 A g^−1^ after 1000 cycles. This finding offers a new perspective for the design of electrodes with large energy storage.

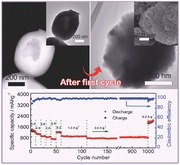

Manganese oxides materials have shown great potential application as energy materials, such as water oxidation and oxygen reduction,^1^, ^2^, ^3^ electrochemical capacitors,^4^, ^5^ lithium–O_2_ batteries,^6^ and lithium‐ion batteries (LIBs).^7^, ^8^, ^9^, ^10^, ^11^, ^12^, ^13^, ^14^, ^15^ Owing to their high specific theoretical capacity, low cost, and environmentally benign nature, manganese oxides (MnO*_x_*, 1 ≤ *x* ≤ 2) are believed to be the most promising alternative anode materials for next generation LIBs.^11^, ^13^, ^16^, ^17^ Moreover, the low operating voltages (1.3–1.5 V for lithium extraction) and small voltage hysteresis (<0.8 V) endow these materials with higher output voltage and higher energy density.^14^, ^15^, ^18^ These two characteristics are important for developing better batteries to meet the increasing demands of our society.^19^ Compared to its counterparts, Mn_2_O_3_ has not been fully investigated, although it features a high theoretical capacity (1018 mAh g^−1^). Currently, the capacity is far lower than its theoretical value and the rate capability is not satisfactory.^8^, ^9^, ^10^ What is more, the fully charged production is ambiguous. As reported by many works, the final oxidation product is MnO*_x_* (*x =* 1 or 1 < *x* < 1.5).^8^, ^12^, ^17^, ^20^ However, nanosized MnO electrode, which features high capacity, could be reoxidated to Mn (IV),^14^, ^21^ and our previous work also confirmed that the nanosized active material possesses enhanced electrochemical kinetics and it is easy to gain a high oxidation product.^22^ Therefore, it is worthwhile to synthesize Mn_2_O_3_ LIBs anode with enhanced capacity and better rate capability by a new route. Meanwhile, gaining further understanding of the conversion mechanism of this material is also an urgent task.

As a characteristic of transition metal oxides electrodes, the nanosize effect is caused by the conversion mechanism (${\mathrm{MO}}_{x}+2x{\mathrm{Li}}^{+}+2xe^{-}M+x{\mathrm{Li}}_{2}O$, M: Mn, Fe, Co, Ni, Cu, etc.), which involves the reversible formation and decomposition of Li_2_O, accompanying the reduction and oxidation of metal (M) nanoparticles (1–5 nm), respectively.^23^ This process induces volume changes and will inevitably destroy the unstable electrode structure, leading to performance degradation.^23^, ^24^ Many merits, however, such as higher specific area, more active sites, and enhanced kinetics of the electrochemical activity, are also induced by the nanosize effect.^25^, ^26^, ^27^, ^28^ As reported by Poizot et al.,^23^ a nanosized electrode was created after the first discharge and preserved on the following charge. This phenomenon is confirmed in many reports.^28^, ^29^ Constructing hollow structure is believed to be an effective way to alleviate the structural strain.^30^, ^31^ However, the conventional hollow structure electrodes with high capacity achieved usually is not with satisfied long cyclic stability. Thus, it is reasonable to design an appropriate structure to improve the electrochemical performance by utilizing these merits and inhibiting the disadvantages simultaneously, achieving high capacity and long cyclic stability.

As multifunctional materials, metal organic frameworks (MOFs) have been used as templates or precursors to fabricate functional materials, recently.^32^ For example, Co_3_O_4_‐carbon nanowire arrays derived from MOFs exhibit high oxygen evolution reaction (OER) activity,^33^ spindle‐like and microboxes α‐Fe_2_O_3_, CuO nanostructures, Zn*_x_*Co_3−*x*_O_4_ hollow polyhedron, and Co_3_O_4_ nanoparticles have been prepared by MOFs and showed high lithium storages.^34^, ^35^, ^36^ However, no reports about the utilization of MnO*_x_* derived from Mn‐based MOFs for LIBs applications, although they have been used for OER.^2^

In this work, polyhedron Mn_2_O_3_ with small interior cavity (mini‐hollow polyhedron Mn_2_O_3_ for short) was derived from Mn‐based MOFs (denoted as Mn‐MOF). Different from the bulk material lacking room to hold the inward volume expansion and the conversional large hollow structure owning too large room for the inward volume expansion without confine, the small interior cavity of a mini‐hollow structure is filled by the reformatted nanoparticles caused by nanosize effect, leading to the formation of a hierarchical nanostructure with homogeneous dispersion of the nanoparticles. When used as LIBs anode material, this mini‐hollow polyhedron Mn_2_O_3_ electrode shows excellent electrochemical performance: high specific capacity, long cycling stability, and superior rate capability (capacity of 819.8 mAh g^−1^ at 1 A g^−1^ after 1200 cycles and 760 mAh g^−1^ at 2 A g^−1^ after 1000 cycles). Further investigations revealed that the nanosize effect plays a key role in improving the electrochemical performance.

Mini‐hollow polyhedrons Mn_2_O_3_ were obtained after the Mn‐MOF being annealed at 750 °C for 4 h with a temperate ramp of 5 °C min^−1^. Typically, 3.75 mmol MnCl_2_·4H_2_O (manganese (II) chloride tetrahydrate) and 10 mmol NH_4_Cl (ammonium chloride) were dissolved in the mixture of 37.5 mL CH_3_CN, 17.5 mL HCOOH, and 17.5 mL CH_3_COOH. The solution was transferred to a 100 mL Teflon‐lined stainless steel autoclave and maintained at 100 °C for 24 h. After cooling down to room temperature naturally, the pink product was collected by suction filtration and washed with ethanol for three times. The pink Mn‐MOF was obtained after drying at 60 °C for 4 h (detailed information can be seen in Figure S1, and Tables S1 and S2 in the Supporting Information). In the calcination process, there is a large temperature gradient, which leads to the transformation of microscale bulk single crystal Mn‐MOF (Figure S1d, Supporting Information) to submicroscale polyhedron manganese oxides (Figures S1e and S2, Supporting Information).^34^ The structure of the product was investigated by X‐ray diffraction (XRD), scanning electron microscope (SEM), and transmission electron microscope (TEM). As is shown in **Figure** **1**a, all the peaks can be well indexed to cubic Mn_2_O_3_ (JCPDS no. 41‐1442).^8^, ^9^, ^37^ Figure 1b,c depicts that the polyhedron Mn_2_O_3_ is well dispersed without any aggregation, reaching a length of ≈0.6–1.2 μm. The smooth surface suggests its single‐crystal character and that is further confirmed by the selected‐area electron diffraction (SAED) patterns in Figure 1e. From a cracked polyhedron (Figure 1c), the inner small hollow space can be clearly observed. The TEM image (inset of Figure 1d and Figure S3a, Supporting Information) with a slight light on the core of polyhedron indicates the small hollow structure. The high‐angle annular dark‐field scanning transmission electron microscope (HAADF‐STEM) image (Figure 1d) confirms the hollow structure with a thick shell and mini‐hollow cavity, different from the conventional hollow structure with a thin shell and large‐hollow cavity.^31^, ^38^, ^39^, ^40^ Obviously, the mini‐hollow cavity could provide the space to hold the inward volume expansion.^13^ The distances of the lattice fringes in Figure 1e are around 2.72 and 3.84 Å, corresponding to the (222) and (211) planes of cubic Mn_2_O_3_.^9^

**Figure 1:**
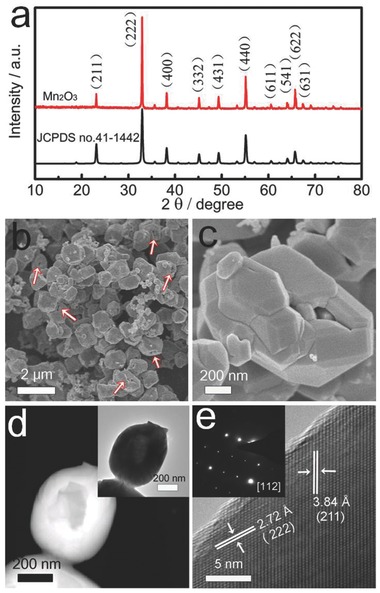
a) XRD of the mini‐hollow polyhedron Mn_2_O_3_, b,c) SEM images, with the arrows in (b) indicating the cracks, d) HAADF‐STEM image, and e) HR‐TEM image. The insets of (d,e) are the corresponding TEM image and SAED patterns.

As **Figure** **2**a shows, the capacity of this mini‐hollow polyhedrons Mn_2_O_3_ electrode shows a slow decrease during the first 60 cycles followed by an increase in subsequent cycles. A capacity of 1370 mAh g^−1^ is stabilized after 450 cycles, exhibiting a high cycling stability and large capacity. All the discharge–charge curves at 0.4 A g^−1^ (Figure 2b) show two plateaus and are highly overlapping, suggesting a two‐step conversion mechanism with high electrochemical reversibility.^41^ The rate capability also is excellent (Figure 2d) and with excellent repeatability (Figure S4a, Supporting Information). At high current densities of 1.0 and 2.0 A g^−1^, stable capacity of 795.3 and 686.7 mAh g^−1^ can be obtained, respectively, and then recovered to 1164.1 mAh g^−1^ at 0.2 A g^−1^. The discharge–charge curves (Figure 2c) are similar at different current densities with limited voltage hysteresis changes, also indicating the high stability of this material.^13^ In addition, the capacity at 2.0 A g^−1^ can be stabilized at ≈760 mAh g^−1^, even after 1000 cycles. What is more, after such an extensive cycling at this aggressive current, an improved capacity of ≈1910 mAh g^−1^ is achieved at 0.2 A g^−1^, which is much larger than both of initial value and the theoretical capacity of Mn_2_O_3_. The over‐theoretical capacity may due to pseudocapacitive charge and partial reversible formation and decomposition of solid‐electrolyte interphase (SEI) layer.^22^, ^28^, ^42^ To the best of our knowledge, this is the best Mn‐based LIBs anodes with such high reversible capacity and super‐long cycling stability (the comparative results are in Table S3, Supporting Information).^11^, ^13^, ^15^, ^17^, ^21^, ^27^, ^41^, ^43^

**Figure 2:**
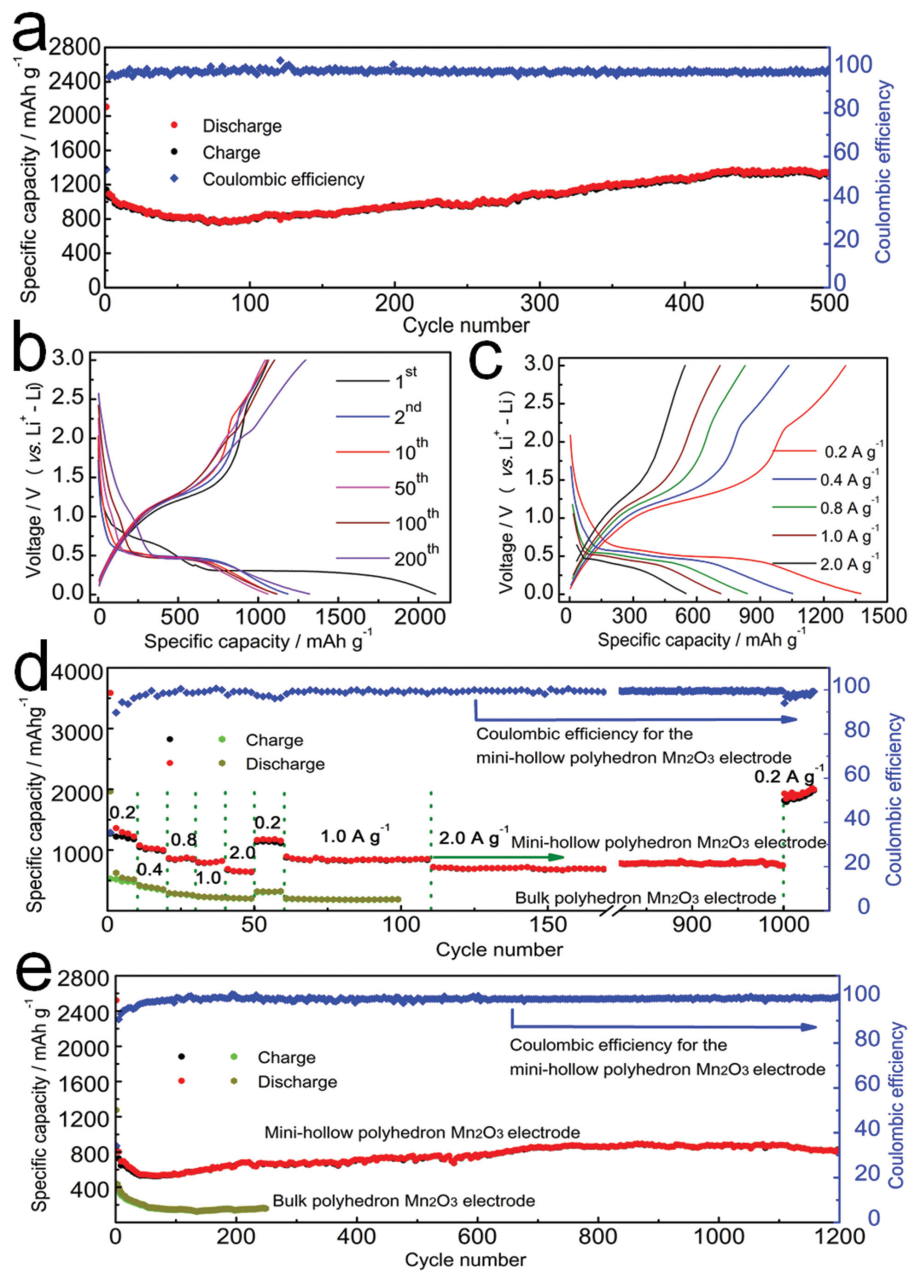
a) Cycling performance and b,c) the corresponding discharge–charge curves at a current density of a,b) 0.4 A g^−1^ and c) various current densities of mini‐hollow polyhedron Mn_2_O_3_ electrode. Cycling performance of the electrodes d) at different current densities and e) a current density of 1.0 A g^−1^.

For a comparison, a bulk polyhedron Mn_2_O_3_ electrode (XRD and SEM are shown in Figure S5, Supporting Information) was prepared.^37^ As is shown in Figure 2e, the mini‐hollow polyhedron Mn_2_O_3_ electrode shows super‐long cycling stability and a capacity of 819.8 mAh g^−1^ is obtained after 1200 cycles at 1.0 A g^−1^, while the bulk polyhedron Mn_2_O_3_ electrode presents a limited cycle performance and only a capacity of ≈160 mAh g^−1^ is retained after 250 cycles (this inferior performance is comparable to reported works).^8^, ^10^ The rate capability (Figure 2d) and the cycling performance at 0.4 A g^−1^ (Figure S4b, Supporting Information) further confirmed this inferior electrochemical performance compared to its mini‐hollow counterpart.

The structural evolution of these two electrodes during cycling was monitored by SEM and the schematics illustration is shown in **Figure** **3**a,b, respectively. In the mini‐hollow polyhedron Mn_2_O_3_ electrode (Figure S6a,b, Supporting Information), a hierarchical nanostructure is formed (as confirmed by TEM in **Figure** **4**c,d) and the small interior cavity disappears after the first cycle. This structural evolution is caused by the nanosize effect of the conversion mechanism and the volume expansion cannot be avoided.^23^ Fortunately, the original small interior cavity offers room for the inward volume expansion, so that it is filled by the reformatted nanoparticles, leading to the formation of a hierarchical nanostructure with homogeneous dispersion of the nanoparticles. This formed hierarchical nanostructure keeps its structure even after 500 cycles (Figure S6c,d, Supporting Information) without any serious “electrochemical sintering,”^14^ indicating its stability. Similarly, the nanosize effects also occur on the bulk polyhedron electrode (Figure S7, Supporting Information). Due to the lack of room to contain the inward volume expansion, the unbalanced expansion tension may lead to the formation of a hierarchical nanostructure with congested core. Obviously, this reconstructed structure is not on equilibrium stage and will aggregate easily and finally collapse. In contrast to our mini‐hollow electrode, the conventional large‐hollow electrodes reported in previous works mostly did not show comparable cyclic stability though their capacity and rate capacity are excellent.^40^, ^44^ As illustrated in Figure 3c, a hollow reconstructed hierarchical nanostructure was formed after the first cycle. Owing to the lack of confine from the interaction with each other, the nanoparticles near the inner cavity may keep expanding and lead to the structure collapse. Thus, those hollow electrodes show limited cycles. This phenomenon can be seen in other conversion mechanism electrodes, such as cobalt oxide.^39^, ^45^ Honestly speaking, the size of the hollow cavity in our system may not be optimal. However, the comparing results indicate that it is important and practical to design and control the hollow size to improve the cycling stability of the conversion mechanism electrodes.

**Figure 3:**
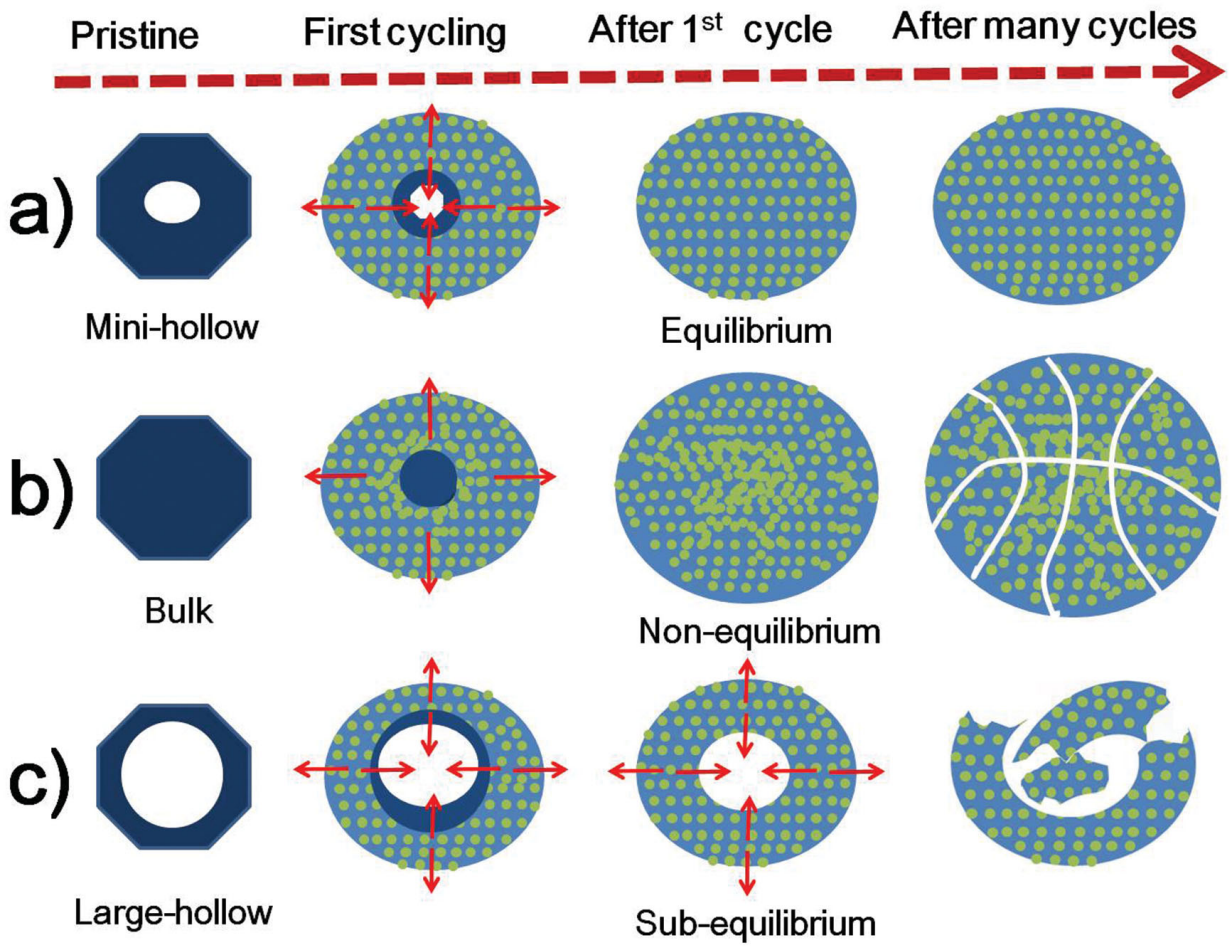
Schematics illustration of the structure evolutions of a) mini‐hollow, b) bulk, and c) large‐hollow polyhedron Mn_2_O_3_ electrodes with cycling.

**Figure 4:**
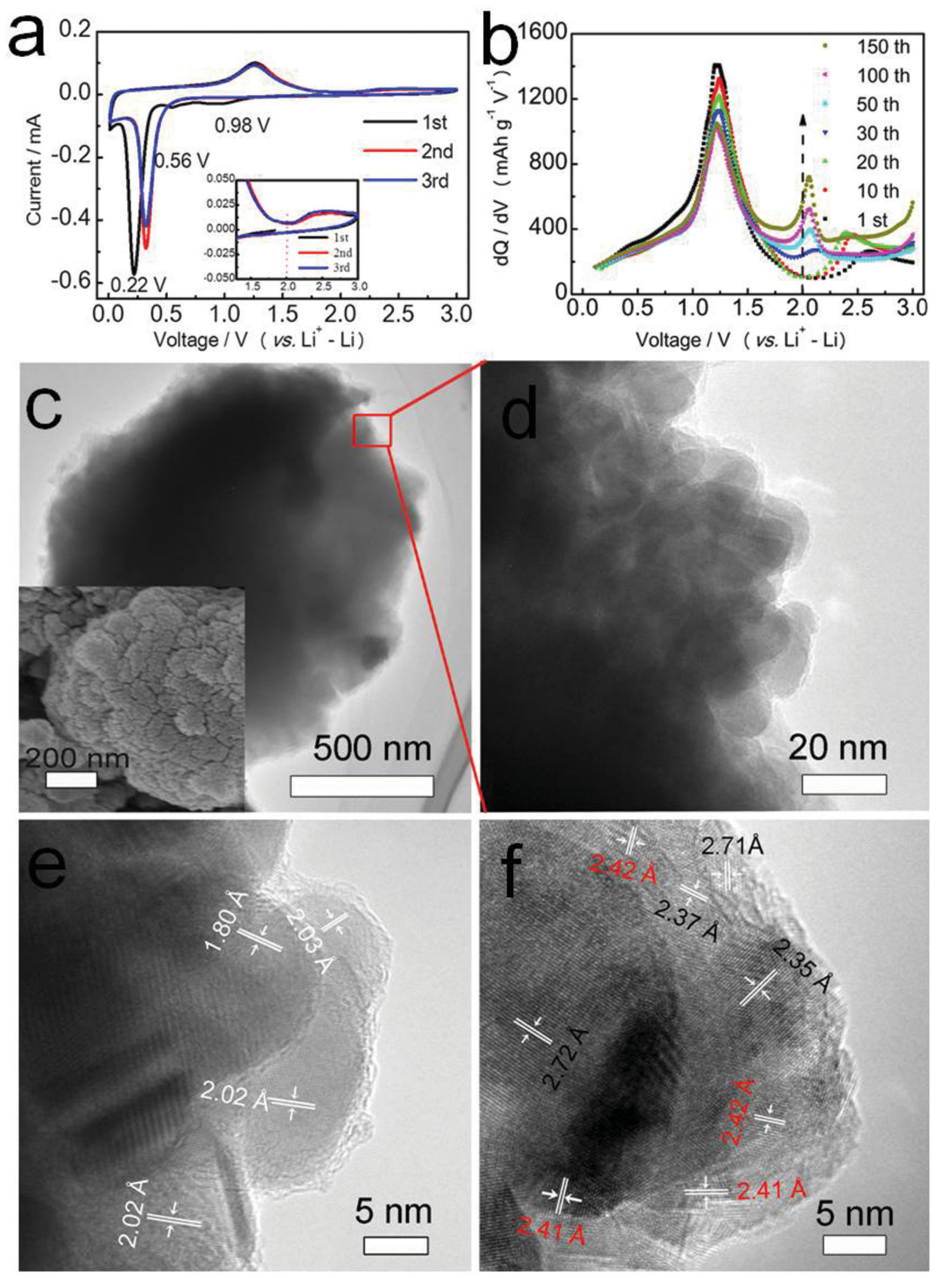
a) CV curves and b) differential charge capacity versus voltage plots of mini‐hollow polyhedron Mn_2_O_3_ electrode. c) TEM, SEM (inset), and d) HR‐TEM of the formed hierarchical nanostructure after the first cycle, e,f) HR‐TEM of the mini‐hollow polyhedron Mn_2_O_3_ electrode at 2.0 and 3.0 V in the first charge. The inset of (a) is the enlarged CV between 1.25 and 3.0 V.

Encouraged by the excellent electrochemical performance of this mini‐hollow Mn_2_O_3_ electrode, a further investigation on its conversion mechanism was carried out. Consisting with many works,^8^, ^10^, ^17^ the peaks located at 0.98 and 0.56 V are considered to be the conversion of Mn(III) to Mn(II) and the formation of the SEI, while the intensive peak at 0.22 V is ascribed to the further reduction of Mn oxide (Mn_3_O_4_) to metallic Mn in the first cathodic process (Figure 4a). However, in contrast to these reported works, there are two peaks located at about 1.25 and 2.5 V on the anodic process, which suggest that the oxidation products at 2.0 and 3.0 V are different. In the high‐resolution TEM (HR‐TEM) image of product at 2.0 V (Figure 4e), only the lattice fringes of Mn_3_O_4_ are observed. After the product is further charged to 3.0 V (Figure 4f), the lattice fringes of Mn_3_O_4_, Mn_2_O_3_, and MnO_2_ are observed (with the *d*‐spacings of lattice fringes for MnO*_x_* (1 < *x* ≤ 2) summarized in Table S4 in the Supporting Information). X‐ray photoelectron spectroscopy (XPS) in Figure S8 (Supporting Information) also confirm the different oxidation states of the products at 2.0 and 3.0 V.^3^, ^4^, ^5^, ^46^ This means that the oxidation peaks at around 1.25 V should be ascribed to the oxidation of metallic Mn to Mn_3_O_4_, and the peak around 2.5 V should be the incomplete oxidation of Mn_3_O_4_ to Mn_2_O_3_ and MnO_2_. The differential charge capacity versus voltage curves (Figure 4b) give further evidence. Obviously, the peak at 2.5 V becomes more and more intensive, shifts to lower potential, finally stabilizes at about 2.1 V. This phenomenon also is reflected by the changes of the two plateaus of the charge curves at 0.4 A g^−1^ (Figure 2b). These results indicate that the oxidation becomes easier as the cycles go on, and the electrochemical kinetics is improved.^14^, ^21^ The subsequent cyclic voltammetry (CV) curves (Figure S9a, Supporting Information) with two pairs of anodic and cathodic peaks give further evidence and the improved kinetics is verified by the reduced charge‐transfer resistance (Figure S9b, Supporting Information).^47^ Based on the above discussion, the lithium storage mechanism of this mini‐hollow polyhedral Mn_2_O_3_ electrode is believed to proceed as follows:

(1) $$\mathrm{First}\;\;\mathrm{discharge}:\;\;{\mathrm{3Mn}}_{2}O_{3}{\mathrm{+2Li}}^{+}{\mathrm{+2e}}^{-}\to {\mathrm{2Mn}}_{3}O_{4}{\mathrm{+Li}}_{2}O$$

(2) $${\mathrm{Mn}}_{3}O_{4}{\mathrm{+8Li}}^{+}{\mathrm{+8e}}^{-}\to \mathrm{3Mn}\;\;+\;\;{\mathrm{4Li}}_{2}O$$

(3) $$\mathrm{Afterward}:\;\;\mathrm{3Mn}\;\;+\;\;{\mathrm{4Li}}_{2}O↔{\mathrm{Mn}}_{3}O_{4}\;\;+\;\;{\mathrm{8Li}}^{+}\;\;+\;\;{\mathrm{8e}}^{-}$$

(4) $$\begin{array}{l} {\mathrm{Mn}}_{3}O_{4}+(3x-4){\mathrm{Li}}_{2}O↔{\mathrm{3MnO}}_{x}+(6x-8){\mathrm{Li}}^{+}\\ \;\;\;\;\;\;\;\;\;\;\;+(6x-8)e^{-}\;(1<x\leq 2) \end{array}$$

In order to further confirm the superior electrochemical property induced by the reconstruction of mini‐hollow structure, the corresponding discharge–charge curves at various current densities, differential charge capacity versus voltage curves, XPS of the products at different charged state, CV curves, and electrochemical impedance spectroscopy (EIS) plots of the bulk polyhedron Mn_2_O_3_ electrode were preformed and presented in Figure S10 (Supporting Information). The discharge–charge curves (Figure S10a,b, Supporting Information) shows that only one pair of plateau on them and the corresponding differential charge capacity versus voltage curves at 0.4 A g^−1^ (Figure S10c, Supporting Information) also shows only one oxidation peak at around 1.25 V and no peaks appears above 2.0 V. The CV curves at different cycles give visualized evidence, and furtherly reveal that the intensity of the peaks lowered with the cycling, as shown in Figure S10d (Supporting Information), suggesting a lowered kinetics. The increased charge‐transfer resistance (Figure S10e, Supporting Information) further evidenced this point. The XPS spectra of the products at 2.0 and 3.0 V stay in the same peak position (Figure S10f, Supporting Information), implying the product at 2.0 V cannot be further oxidated in this bulk electrode. All these evidences show that the bulk Mn_2_O_3_ electrode owes a sluggish kinetic compared with the mini‐hollow Mn_2_O_3_ electrode.

Based on the above analysis, the outstanding electrochemical properties exhibited by this mini‐hollow polyhedron Mn_2_O_3_ electrode are mainly induced by the nanosize effect by utilizing its merits and inhibiting its disadvantages simultaneously. First, the small interior cavity offers room for the inward volume expansion, forming a hierarchical nanostructure with homogeneous dispersion of the reformatted nanoparticles. Second, the reconstructed hierarchical nanostructure after the first cycle remains steady for long cycling stability. Third, the nanostructure not only induces more active sites to join in the electrochemistry activity but also enhances the kinetics to easily produce higher oxidation products, achieving a large capacity and good rate capability.

In summary, mini‐hollow polyhedron Mn_2_O_3_ derived from Mn‐based MOFs has been synthesized. This material exhibits promising Li storage property by utilizing the nanosize effect ingeniously. A high capacity and excellent rate capability were achieved. Meanwhile, the reasons for the improved electrochemical activities were studied and a new mechanism is proposed. What is more, the super‐long cycling stability (exceeding 1200 cycles at 1.0 A g^−1^) and high capacity endow this material with a competitive prospects for application. The discussion on the mini‐hollow structure suggests that it is important and practical to design and control the hollow size to improve the cycling stability of conversion mechanism electrodes, which offers a new perspective to design the structure of an electrode material with high‐performance energy storage.

## Experimental Section

All the chemicals were purchased from J&K and used without further purification.

*Materials Synthesis*: Typically, 3.75 mmol MnCl_2_·4H_2_O and 10 mmol NH_4_Cl were dissolved in the mixture of 37.5 mL CH_3_CN (acetonitrile), 17.5 mL HCOOH (methanoic acid), and 17.5 mL CH_3_COOH (acetic acid). The solution was transferred to a 100 mL Teflon‐lined stainless steel autoclave and maintained at 100 °C for 24 h. After cooling down to room temperature naturally, the pink product was collected by suction filtration and washed with ethanol for three times. The pink Mn‐based MOF was obtained after drying at 60 °C for 4 h. In order to obtain mini‐hollow polyhedron Mn_2_O_3_, the prepared Mn‐based MOF was heated at 750 °C with a temperate ramp of 5 °C min^−1^ for 4 h in air.

The bulk polyhedron Mn_2_O_3_ was synthesized according to the reported work.^37^ Typically, 16 mmol of Mn(NO_3_)_2_ was dissolved in 50 mL 1‐butanol solvent followed by a vigorous stirring for half an hour at room temperature. Then the mixture was transferred to a 100 mL Teflon liner and sealed in an autoclave for solvothermal treatment at 120 °C for 20 h. The black bulk polyhedron Mn_2_O_3_ was collected by suction filtration and washed with ethanol for three times.

*Single‐Crystal X‐Ray crystallography*: Suitable crystal Mn‐MOF was placed in a cooled N_2_ gas stream at ≈130 K for crystallographic data collection on a SuperNova Single Crystal Diffractometer equipped with graphite‐monochromatic Mo Kα radiation (*λ* = 0.71073 Å). Data reduction included absorption was performed by using the SAINT program.^48^ The structures were solved by direct methods and refined by full‐matrix least squares on F2 with SHELXS‐97 and SHELXL‐97 programs.^49^

*Materials Characterization*: The crystal structures of the products were characterized by XRD (Rigaku MiniFlexII, with Cu Kα radiation, *λ* = 1.5408 Å), The morphology and microstructure were characterized by scanning electron microscopy (SEM; JEOL JSM‐7500F) and transmission electron microscopy (TEM; Philips Tecnai G2 F20). Thermogravimetric analysis (NETZSCH, TG209) was carried out under air flow with a temperature ramp of 5 °C min^−1^. Elemental analyses (for C, N, and H) were performed by using an Elementar analyzer (vario EL CUBE). The binding energy of Mn was investigated by XPS (Kratos Axis Ultra DLD spectrometer).

*Electrochemical Measurements*: Electrochemical measurements were carried out using a two‐electrode cell assembled in an argon‐filled glove box. The working electrodes consist of 75 wt% active material (Mn_2_O_3_), 15 wt% Super P carbon black, and 10 wt% sodium carboxymethyl cellulose. The loading amount of the electrode material was measured ≈0.60 mg cm^−2^ by a microbalance (Mettler, XS105DU) with an accuracy of 0.01 mg. The electrolyte is 1 m LiPF_6_ in a mixture of ethylene carbonate (EC) and diethyl carbonate (DMC) (EC:DMC = 1:1 by volume). Pure lithium foil was used for both the counter and reference electrode. And the separator was Celgard 2320 membrane. CV testing with a cutoff voltage window of 0.01–3.00 V (vs Li^+^–Li, 0.1 mV s^−1^) and EIS (0.1–100k Hz) measurements were both performed on a CHI660b electrochemical workstation (Chenhua, Shanghai, China). Galvanostatic charge–discharge tests were carried out on a Land Battery Measurement system (Land CT2001A, Wuhan, China) under various current densities in the fixed range of 3.00–0.01 V at room temperature.

## Supporting information

As a service to our authors and readers, this journal provides supporting information supplied by the authors. Such materials are peer reviewed and may be re‐organized for online delivery, but are not copy‐edited or typeset. Technical support issues arising from supporting information (other than missing files) should be addressed to the authors.

Supplementary
