# Supplementary material for: Reconstruction of Mini‐Hollow Polyhedron Mn2O3 Derived from MOFs as a High‐Performance Lithium Anode Material
Source: Adv Sci (Weinh). 2015 Aug 25;3(3):1500185. doi: 10.1002/advs.201500185 (PMC5049611; doi:10.1002/advs.201500185)
Supplement: Supplementary file 1 — Supplementary [file ADVS-3-1500185-s001.pdf]

Copyright WILEY-VCH Verlag GmbH & Co. KGaA, 69469 Weinheim, Germany, 2015.

ADVANCED SCIENCE
Open Access

Supporting Information

for *Adv. Sci.*, DOI: 10.1002/advs. 201500185

Reconstruction of Mini-Hollow Polyhedron Mn_2O_3 Derived from MOFs as a High-Performance Lithium Anode Material

Kangzhe Cao, Lifang Jiao, Hang Xu, Huiqiao Liu, Hongyan Kang, Yan Zhao, Yongchang Liu, Yijing Wang,* and Huatang Yuan*

Copyright WILEY-VCH Verlag GmbH & Co. KGaA, 69469 Weinheim, Germany, 2013.

Supporting Information

Reconstruction of Mini-hollow Polyhedron Mn_2O_3 Derived from MOFs as High-Performance Lithium Anode Material

Kangzhe Cao, Lifang Jiao, Hang Xu, Huiqiao Liu, Hongyan Kang, Yan Zhao, Yongchang Liu, Yijing Wang,* and Huatang Yuan*

E-mail: jiaolf@nankai.edu.cn; wangyj@ nankai.edu.cn

CONTENTS:

Part 1: Tables (S1-S4)

Part 2: Figures (S1-S10)

Part 1: Tables (S1-S4)

Table S1. Crystal data and structure refinement for Mn-MOF.

Parameter	Identification code Mn-MOF
Empirical formula	$CH_5Cl_2MnNO_2$
Formula weight	188.90
Temperature/K	126.30(10)
Crystal system	monoclinic
Space group	C2/c
a/Å	7.8665(5)
b/Å	11.4083(7)
c/Å	6.9697(4)
α/°	90.00
β/°	108.458(7)
γ/°	90.00
Volume/Å^3	593.31(6)
Z	4
ρ_{calc}g/cm^3	2.115
μ/mm^{-1}	3.017
F(000)	372.0
Crystal size/mm^3	? × ? × ?
Radiation	MoKα (λ = 0.71073)
2Θ range for data collection	6.52 to 50.02
Index ranges	$-9 \le h \le 8$, $-8 \le k \le 13$, $-7 \le l \le 8$
Reflections collected	1044
Independent reflections	530 [R_{int}= 0.0562, R_{sigma}= 0.0782]
Data/restraints/parameters	530/0/36
Goodness-of-fit on F^2	1.016
Final R indexes [I>=2σ (I)]	R_1= 0.0549, wR_2= 0.1708
Final R indexes [all data]	R_1= 0.0607, wR_2= 0.1774
Largest diff. peak/hole / e Å^{-3}	0.71/-0.67

Table S2. The contents of C, N and H elements in the Mn-MOF

Parameter	C (%)	N (%)	H (%)
Experimental value	6.55	7.79	2.62
Theoretical value	6.35	7.41	2.65

Table S3. Specific capacities and cycling properties of MnO_x as anodes for LIBs

Materials	Capacity(mA h g^{-1}) / Current density (A g^{-1})		Reference
Mn_3O_4@graphene	730 / 0.4 (40th)	390 / 1.6 (35th)	[1]
MnO@CNT	1082 / 0.1 (100th)	575 / 1.0 (200th)	[2]
MnOx@Carbon	650 / 0.2 (130th)	500 / 0.8 (150th)	[3]
MnO/C nanopeapods	1119 / 0.5 (100th)	463 / 5.0 (40th)	[4]
MnO/C Networks	1224 / 0.2 (200th)	730 / 1.5 (200th)	[5]
MnO2 /CNT	620 / 0.2 (50th)	420 / 2.0	[6]
Porous Mn_2O_3 Nanoplates	813.7 / 0.1 (50th)	390 / 2.0 (25th)	[7]
porous Mn_2O_3 nanomaterial	521 / 0.3 (100th)	500 / 0.5 (60th)	[8]
Mn_2O_3 NWs on Ti foil	~ 500 / 0.1 (100th)	220 / 1.0 (120th)	[9]
hollow Mn_2O_3 spheres	~750 / 0.2 (100th)	470 / 3.2 (100th)	[10]
Mn_3O_4 hollow spheres	980 / 0.2 (140th)	750 / 2.0 (40th)	[11]
Mn_2O_3 microspheres	524 / 0.2 (200th)	155 / 1.0 (1000th)	[12]
Mn_3O_4 hollow spheres	1165 / 0.1 (60th)	690 / 1.0 (50th)	[13]
**Mini-hollow polyhedron Mn_2O_3**	**1164 / 0.2 (60th)**<br>**1370 / 0.4 (500th)**	**819 / 1.0 (1200th)**<br>**760 / 2.0 (1000th)**	**This work**

Table S4. The *d*-spacings and the corresponding Miller index of the MnO_x at 2.0 and 3.0 V

d-spacing experimental value (Å)	Phase	*d*-spacing reported value (Å)	Miller index	Ref. (JCPDS)
1.80	Mn_3O_4	1.79	(105)	2-1062
2.02	Mn_3O_4	2.03	(220)	2-1062
2.35/2.37	Mn_3O_4	2.35	(004)	2-1062
	Mn_2O_3	2.35	(400)	41-1442
2.41/2.42	MnO_2	2.42	(311)	42-1169
2.71/2.72	Mn_2O_3	2.72	(111)	41-1442

Part 2: Figures (S1-S10)

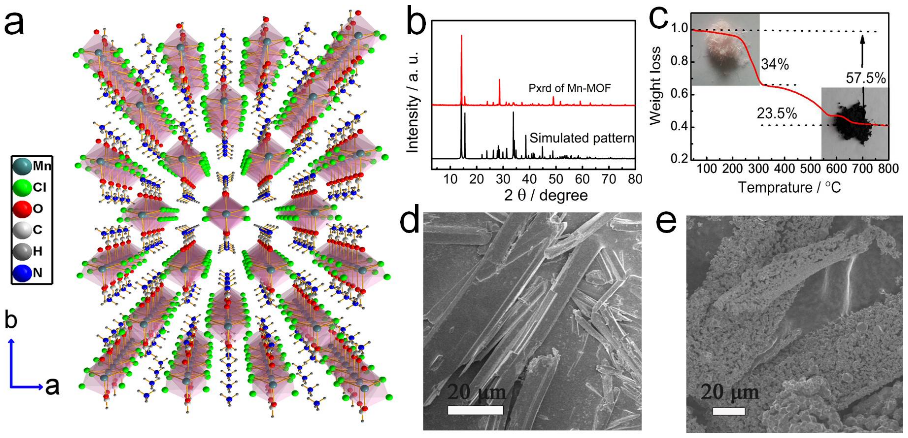

Figure S1. Crystal structure (a) and Powder XRD pattern (b) of Mn-MOF; (c) TG curves of Mn-MOF in air with a temperature ramp of 5 °C min^{-1}. (d) and (e) are the SEM images of the Mn-MOF before and after being calculated at 750 °C in air for 4 h. Inset of (c) is the picture of Mn-MOF(pink) and its annealed product Mn_2O_3 (black).

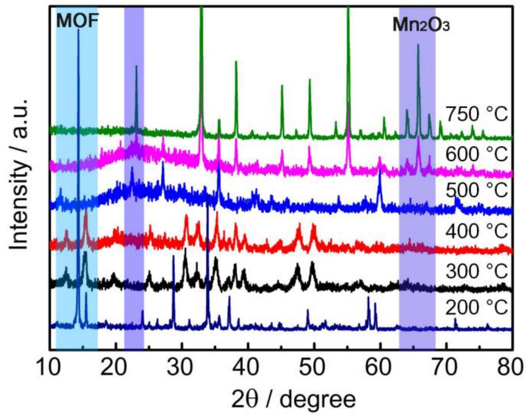

Figure S2. The XRD patterns of the annealed Mn-MOF products at different temperatures in air for 4 h. The typical peaks in blue section are for Mn-MOF and that in purple sections are for Mn_2O_3.

As revealed by the Single-crystal X-ray crystallography data (Table S1) and Figure S1a, each Mn2$^+$ atom in Mn-MOF is six-coordinated to two oxygen atoms from two $HCOO^-$ and four Cl^-. The adjacent Mn2$^+$ is linked by $HCOO^-$ and Cl^- to form the one-dimensional channels with a Mn-Mn distance of 3.485 Å along the c-direction. empirical formula of the prepared Mn-MOF is $CH_5Cl_2MnNO_2$ and its molecule formula is $MnCl_2COOH{\cdot}NH_4$. This result is further confirmed by thermogravimetric analysis (TGA) and Elemental analyses (EA)

(as is shown is Figure S1 and Table S2). The TG curve of this Mn-MOF precursor showed a total weight loss of 57.5% when heating to 720 °C, in accordance with the theoretical value 58.2%. The first weight loss of 34% from room temperature to 300 °C can be attributed to the removal of guest molecule NH_4^+ and $COOH^-$, and the second main weight loss of 23.5% from 300 °C to 720 °C is most likely owing to thermal transform of the rest into Mn_2O_3. [14]

The phases of the products annealed at different temperatures were monitored by X-ray diffraction (XRD, Figure S2). It revealed that the Mn-MOF phase totally disappeares above 500 ºC, while the phase and crystallinity of Mn_2O_3 increased with increasing annealed temperature. After the Mn-MOF was annealed at 750 ºC for 4h, submicroscale mini-hollow polyhedrons Mn_2O_3 were obtained.

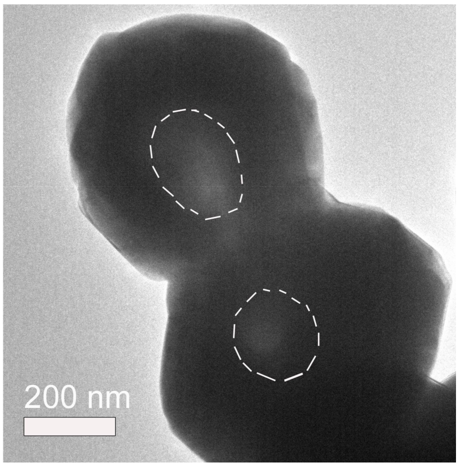

Figure S3. TEM image of mini-hollow polyhedron Mn_2O_3.

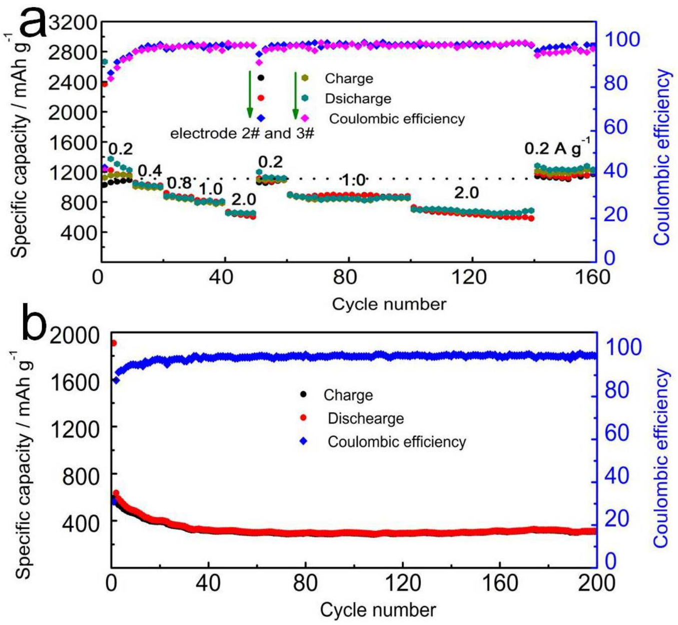

Figure S4. (a) Rate capabilities of another two mini-hollow polyhedron Mn_2O_3 electrode and (b) cycling performance of bulk polyhedron Mn_2O_3 at 0.4 A g^{-1}.

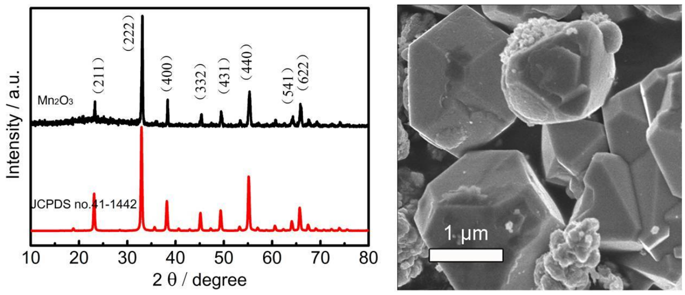

Figure S5. (a) The XRD pattern and (b) SEM image of bulk polyhedron Mn_2O_3.

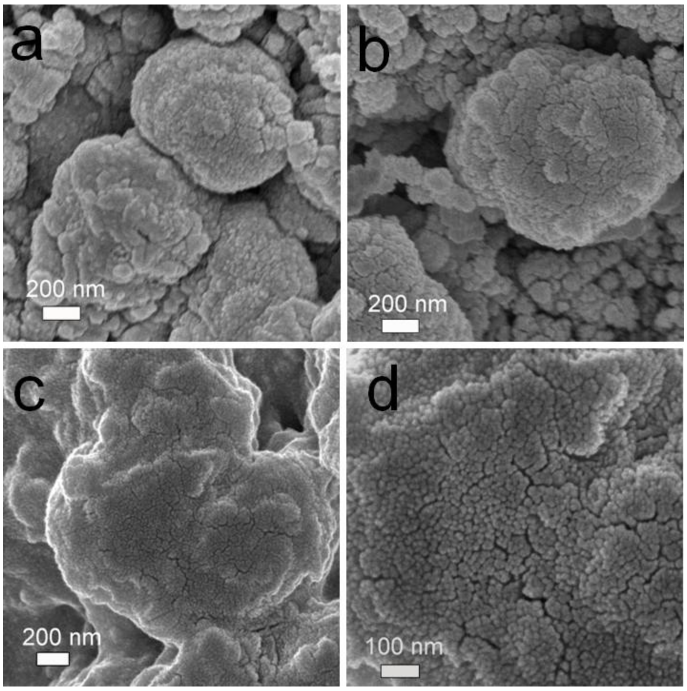

Figure S6. SEM images of the mini-hollow polyhedron Mn_2O_3 electrode after 3 cycles (a), 50 cycles (b) and 500 cycles (c, d).

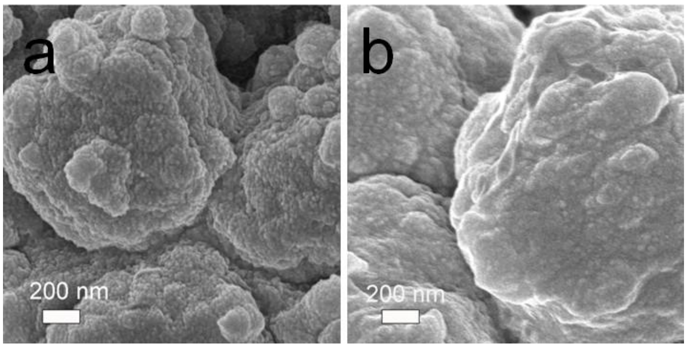

Figure S7. SEM images of the buck polyhedron Mn_2O_3 electrode after (a)3 cycles, (b) 50 cycles.

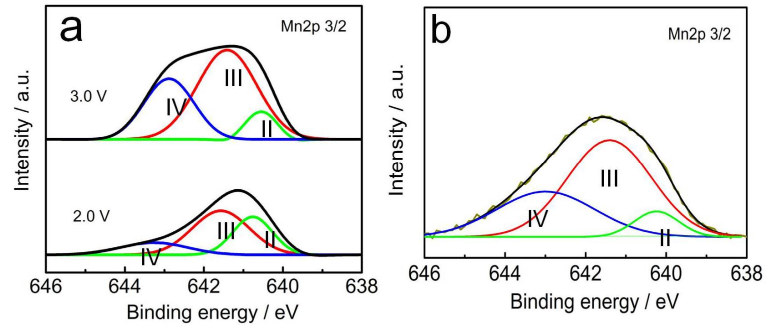

Figure S8. (a) The XPS spectra in different charge state (2.0 V and 3.0 V) and (b) XPS depth profiles in 3.0 V charge state with the argon etching of the surface of the mini-hollow polyhedron Mn_2O_3 electrode.

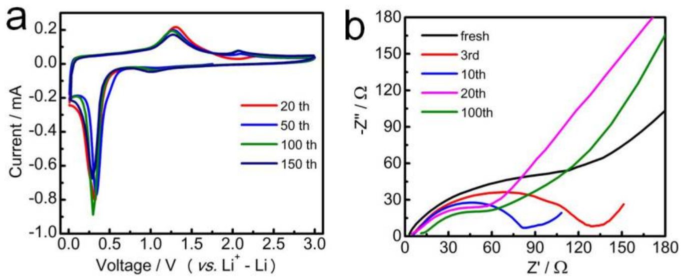

Figure S9. The CV (a) and EIS (b) of the mini-hollow polyhedron Mn_2O_3 electrode after different cycles.

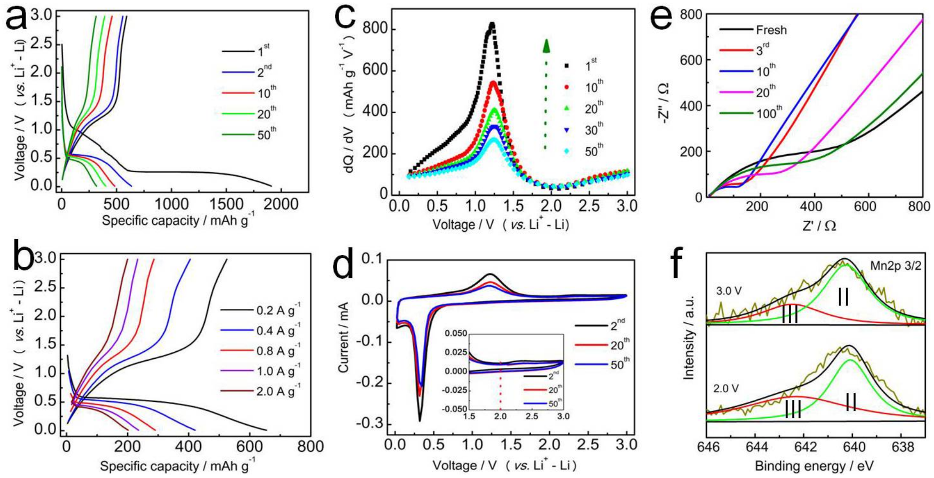

Figure S10. The electrochemical performance of bulk polyhedron Mn_2O_3 electrode. Discharge-charge curves at 0.4 A g^{-1} (a) and various current densities (b), differential charge capacity *vs*. voltage plots (c), CV curves (d) and EIS plots (e). XPS spectra of the bulk polyhedron Mn_2O_3 electrode charged products at 2.0 V and 3.0 V.

Reference:

[1] H. L. Wang, L. F. Cui, Y. Yang, H. S. Casalongue, J. T.Robinson, Y. Y. Liang, Y. Cui, H. J. Dai, *J. Am. Chem. Soc.* **2010**, *132*, 13978.

[2] B. Liu, X. L. Hu, H. H. Xu, W. Luo, Y. M. Sun, Y. H. Huang, *Sci. Rep.* **2014**, *4*, 4229.

[3] J. C. Guo, Q. Liu, C. S. Wang, M. R. Zachariah, *Adv. Funct. Mater.* **2012**, *22*, 803.

[4] H. Jiang, Y. J. Hu, S. J. Guo, C. Y. Yan, P. S. Lee, C. Z. Li, *ACS Nano* **2014**, *8*, 6038.

[5] W. Luo, X. L. Hu, Y. M. Sun, Y. H. Huang, *ACS Appl. Mater. Interfaces* **2013**, *5*, 1997.

[6] H. Xia, M. Lai, L. Lu, *J. Mater. Chem.* **2010**, *20*, 6896.

[7] Y. H. Zhang, Y. Yan, X. Y. Wang, G. Li, D. R. Deng, L. Jiang, C. Y. Shu, C. R. Wang, *Chem. Eur. J.* **2014**, *20*, 6126.

[8] X. Zhang, Y. T. Qian, Y. C. Zhu, K. B. Tang, *Nanoscale* **2014,** *6*, 1725.

[9] Y. H. Wang, D. Jia, Z. Peng, Y. Y. Xia, G. F. Zheng, *Nano Lett.* **2014**, *14*, 1080.

[10] Y. Qiao, Y. Yu, Y. Jin, Y. B. Guan, C. H. Chen, *Electrochimica Acta* **2014**, *132*, 323.

[11] G. Jian, Y. H. Xu, L. C. Lai, C. S. Wang, M. R. Zachariah, *J. Mater. Chem. A* **2014**, *2*, 4627.

[12] L. Chang, L. Q. Mai, X. Xu, Q. Y. An, Y. L. Zhao, D. D. Wang, X. Feng, *RSC Adv.* **2013**, *3*, 1947.

[13] J. Yue, X. Gu, L. Chen, N. N. Wang, X. L. Jiang, H. Y. Xu, J. Yang, Y. T. Qian, *J. Mater. Chem. A* **2014,** *2*, 17421.

[14] Y. C. Qiu, G. L. Xu, K. Y. Yan, H. Sun, J. W. Xiao, S. H. Yang, S. G. Sun, L. M. Jin, H. Deng, *J. Mater. Chem.* **2011**, *21*, 6346.
